# Systematic Construction and Validation of an RNA-Binding Protein-Associated Prognostic Model for Acute Myeloid Leukemia

**DOI:** 10.3389/fgene.2021.715840

**Published:** 2021-09-24

**Authors:** Hongwei Luo, Yingchun Zhang, Nan Hu, Yancheng He, Chengcheng He

**Affiliations:** ^1^People’s Hospital of Mianzhu, Deyang, China; ^2^People’s Hospital of Zhongjiang, Deyang, China; ^3^Southwest Medical University, Luzhou, China; ^4^Jiangyang City Construction College, Luzhou, China

**Keywords:** acute myeloid leukemia, RNA-binding proteins, prognostic signature, bioinformatics, prognostic model

## Abstract

**Background:** The abnormal expression of RNA-binding proteins (RBPs) in various malignant tumors is closely related to the occurrence and development of tumors. However, the role of RBPs in acute myeloid leukemia (AML) is unclear.

**Methods:** We downloaded harmonized RNA-seq count data and clinical data for AML from UCSC Xena, including The Cancer Genome Atlas (TCGA), The Genotype-Tissue Expression (GTEx), and Therapeutically Applicable Research to Generate Effective Treatments (TARGET) cohorts. R package *edgeR* was used for differential expression analysis of 337 whole-blood data and 173 AML data. The prognostic value of these RBPs was systematically investigated by using univariate Cox regression analysis, least absolute shrinkage and selection operator (LASSO)–Cox regression analysis, and multivariate Cox regression analysis. C-index and calibration diagram were used to judge the accuracy of the model, and decision curve analysis (DCA) was used to judge the net benefit. The biological pathways involved were revealed by gene set enrichment analysis (GSEA). The Gene Ontology (GO) and Kyoto Encyclopedia of Genes and Genomes (KEGG) pathway analysis and the protein–protein interaction (PPI) network performed lateral verification on the selected gene set and LASSO results.

**Results:** A prognostic model of 12-RBP signature was established. In addition, the net benefit and prediction accuracy of the prognostic model and the mixed model based on it were significantly higher than that of cytogenetics. It is verified in the TARGET cohort and shows good prediction effect. Both the selection of our gene set and the LASSO results have high credibility. Most of these pathways are involved in the development of the disease, and they also accumulate in leukemia and RNA-related pathways.

**Conclusion:** The prognosis model of the 12-RBP signature found in this study is an optimized biomarker that can effectively stratify the risk of AML patients. Nomogram based on this prognostic model is a reliable method to predict the median survival time of patients. This study expands our current understanding of the role of RBPs in the occurrence of AML and may lay the foundation for future treatment of the disease.

## Introduction

Acute myeloid leukemia (AML) is a cancer of myeloid blood cells, characterized by clonal dilatation of myeloid precursors at different stages of differentiation, resulting in dysgenesis of normal blood cells, and bone marrow failure (Board, 2019). It is the most common subtype of leukemia, with genetic diversity, a worldwide incidence of 3/100,000 per year, poor prognosis, and high mortality (Zhou and Chng, 2014; Döhner et al., 2015). Although most AML patients achieve their first complete remission after induction therapy, relapse is the main reason for the high mortality of patients (Ravandi, 2013; Cornelissen et al., 2015). Postremission therapy (PRT) is an important means to prevent recurrence. According to the recommendations of the European LeukemiaNet (ELN), patients at adverse risk should opt for allogeneic transplantation, and those at favorable molecular risk should undergo intensive chemotherapy (Döhner et al., 2017). Thus, prognostic assessment of the patient is critical to the development of appropriate treatment decisions and follow-up strategies. Cytogenetics is an important prognostic factor for AML and is the basis of current risk classifications for the disease (Döhner et al., 2010; Grimwade et al., 2010). Many cytogenetic abnormalities are known to be associated with poor prognosis and a higher risk of relapse after treatment (Slovak et al., 2000). However, some patients still relapse in the absence of adverse risk factors (Röllig et al., 2011). Therefore, in order to improve the prognosis assessment of AML patients, biomarkers must be optimized. Current sequencing work has revealed extensive genomic heterogeneity of AML and provided valuable information on diagnosis and prognosis, and enabling the optimization of biomarkers.

RNA-binding proteins (RBPs) are proteins that interact with RNA through RNA-binding domains. As important coordinators for maintaining genomic integrity, RBPs are widely expressed in cells and play a core and conservative role in gene regulation (Gerstberger et al., 2014; Nishida et al., 2017). RBPs are involved in regulating all aspects of RNA metabolism and function, including RNA biogenesis, maturation, transport, cellular localization, and stability (Masuda and Kuwano, 2019). When the nuclear RNA emerges from the RNA polymerase, the RNA transcript is immediately covered by the RNA-binding protein, exerting its functions and ultimately affecting the expression of each gene (Campos-Melo et al., 2014). Given the importance of RBPs in regulating life processes, it is not surprising that some aberrant, deregulated RBPs are closely associated with the onset and progression of disease.

Because of their important role, RBPs have been widely studied in recent years. RBPs have been found to play a critical role in tumor development, and hundreds of RBPs are clearly dysregulated in cancer (Wang et al., 2018). In fact, previous studies have linked known cancer drivers to RBP disorders, including AML. Some RBP-encoding genes promote the development of cancer cells. For example, TRIM21 promotes the transformation of breast cancer cells from epithelium to stroma (Jin et al., 2019); FOXK2 promotes colorectal cancer metastasis by upregulating ZEB1 and EGFR expression (Du et al., 2019). Wang E. et al. (2019) revealed 21 RBP candidates upregulated in AML that are critical for maintaining RNA splicing and survival of AML. Mutations in RPS14, SRBP2, SF3B1, and U2AF1 can lead to myelodysplastic abnormalities, hematopoietic dysfunction, AML, and other blood-related diseases (Ebert et al., 2008; Komeno et al., 2015; Shirai et al., 2015; Mortera-Blanco et al., 2017; de Rooij et al., 2019).

Taken together, these studies suggest that RBPs is closely related to the occurrence and development of human tumors. RBP-encoding genes have been used to build prognostic models of cancer but are still lacking in AML. For example, Li et al. (2020) used eight-RBP gene to predict the prognosis of patients with lung adenocarcinoma. Therefore, in AML, systematic use of high-throughput transcriptome data to identify the expression profile of RBP-encoding genes in normal and tumor tissues is a necessary step to understand its role in the pathogenesis, which not only contributes to the understanding of the pathogenesis, but also has a guiding role in the prognosis.

## Materials and Methods

### Data Collection

UCSC Toil RNA-seq Recompute^1^ processing more than 20,000 unaligned RNA samples from The Cancer Genome Atlas (TCGA),^2^ Therapeutically Applicable Research to Generate Effective Treatments (TARGET),^3^ and Genotype-Tissue Expression (GTEx)^4^ datasets resulted in a combined cohort free of computational batch effects between different repository. In this study, the RNA-seq expression profiles and corresponding clinical data of AML patients in the TCGA and TARGET were retrieved, respectively, from the UCSC Toil RNA-seq Recompute. The AML patients from TCGA-LAML project were chosen as the training cohort to establish the risk classification system based on the RBP signatures and to construct predictive model. An independent dataset (TARGET^5^) was employed for its external validation. The case selection criteria for data extraction were patients diagnosed with AML and available clinical information such as survival status and overall survival time, age, gender, FAB classification, and cytogenetic risk stratification.

For RBP-encoding genes, we obtained a reliable correlation gene summarized by Gerstberger et al. (2014)^6^. The summation of all RBP-encoding genes was used to further identify AML-related features. For clinical characteristics, R package *tableone* was used to use chi-square test for classified data, and analysis of Kruskal test was used for continuous variables, which were represented by median (Yoshida et al., 2020).

### Identification of Differentially Expressed mRNA in Acute Myeloid Leukemia

Differential expression analysis was performed between 337 whole blood RNA-seq data of GTEx and 173 AML RNA-seq data of samples of TCGA using the R package *edgeR* (Robinson et al., 2010), and the screening criteria were |log2 (fold change)| ≥ 1.5 and FDR <0.05. The final results were visualized by using *ggplot* to plot the volcano for the differences between RBP-encoding genes and remaining genes (Wickham, 2016).

### Construction of the Prognostic RNA-Binding Protein-Encoding Gene Signature

Univariate Cox regression analysis and Least absolute shrinkage and selection operator (LASSO)-Cox regression analysis were performed to identify the prognosis-related RBP-encoding genes and construct the prognostic gene signature. We used the TCGA data set as the training cohort and the TARGET data set as the validation cohort.

Least absolute shrinkage and selection operator is a popular method that avoids overfitting by incorporating the best performance parameters to produce a simpler and more easily interpreted model, which is widely used in Cox proportional hazard regression model for high-dimensional data survival analysis (Simon et al., 2011). The R package *survival* was used for Univariate Cox regression analysis, and the RBP-encoding genes with differential expression of *p* < 0.01 were screened and incorporated into the LASSO regression model (Therneau and Grambsch, 2000). The LASSO regression was analyzed with R package *glmnet*, and the prognosis model of RBP-encoding genes was generated (Friedman et al., 2010). In the LASSO regression, the setting parameters are cross-verified, and the partial likelihood deviation satisfies the minimum criterion. The risk score was constructed based on the expression of prognostic RBP-encoding genes. The risk score for each sample was calculated as the following formula:


$$Risk\;score\;=\sum_{i}(Coef_{i}\times Exp_{i})$$

***Exp*_*i*_** is the relative expression of the gene in the patient signature, and ***Coef*_*i*_** was the LASSO coefficient of the gene. The median risk score in the training cohort was used as the cutoff value for the AML cohort dichotomy. In both the training and validation sets, patients were divided into high- and low-risk groups based upon resultant risk score values, respectively. Kaplan–Meier (KM) survival curve and time-dependent ROC curve estimates were then performed for each cohort to assess the predictive power of the prognostic model of RBPs.

### Model Construction and Validation

To determine whether genes can be used independent of clinical information as a prognostic indicator for AML patients, univariate and multivariate Cox regression analyses were performed.

Univariate Cox screening index was used, which included the clinical characteristics common to TCGA cohort and the TARGET cohort, as well as the high- and low-risk index of base 12 gene construction, to include the index with *p* < 0.05 into the multivariate Cox regression model. Then stepwise regression was employed to further select the best model. Forest plots provided a visualization of the hazard ratio (HR) and 95% confidence intervals.

The nomogram is a kind of visual regression model, which sets the scoring standard according to the regression coefficient of independent variables, through which we can calculate and predict the patient outcome by comparing the situation of the patient (Iasonos et al., 2008). In our study, a combined model of all independent prognostic characteristics screened by regression analysis was used to establish a nomogram to assess median survival in AML patients. The calibration diagram and C index are used to evaluate the predicted results of the regression model. The decision curve analysis (DCA) quantifies the net benefit under different threshold probabilities to determine the clinical practicability of nomograms and to find the model that predicts the maximum net benefit, so it is widely used (Vickers and Elkin, 2006). The C index and DCA are used to compare the prediction accuracy between individual components and composite models.

### Protein–Protein Interaction, Gene Ontology, and Kyoto Encyclopedia of Genes and Genomes Analyses Were Used to Further Verify the Results

After differential analysis, differentially expressed RBP-encoding genes were extracted for Gene Ontology (GO) and Kyoto Encyclopedia of Genes and Genomes (KEGG) analyses to determine whether they were enriched in RBP-related pathways. We constructed a protein–protein interaction (PPI) network for the different RBP-encoding genes, identified the core module, and judged the distribution of genes screened by LASSO in the module. In the GO analysis and KEGG pathway analysis, respectively, enrichGO function and enrichKEGG function in the R package *clusterProfiler* are used (Yu et al., 2012). Finally, the bubble chart showing both was used (Yu et al., 2012). Both *p*-values and *q*-values <0.05 were considered statistically significant. We input the differentially expressed RBP-encoding genes into the STRING database^7^ to obtain the protein–protein interaction network, and the results were visualized using Cytoscape 3.7.2 software (Franceschini et al., 2012). The molecular complex detection (MCODE) plug-in was used to identify the core module, and the distribution of the 12-RBP signature in the core module was detected, and then the detected module was displayed.

### Gene Set Enrichment Analyses

Gene set enrichment analysis (GSEA) is used to determine whether a defined set of genes has statistically significant and consistent differences between two biological states (Subramanian et al., 2005). Using the R package *clusterProfiler* (Yu et al., 2012), the potential biological pathways between high- and low-risk groups were recognized. The enriched pathways in each phenotype were sequenced using nominal *p*-values and normalized enrichment scores (NES), and we selected a subset of these pathways for display.

## Results

### Patient Characteristics

As shown in Table 1, a total of 151 TCGA cohort samples and 228 TARGET cohort samples were divided into high- or low-risk groups by 12 prognostic RBP-encoding genes, and the data distribution of different clinical characteristics and statistical tests was calculated. In the TCGA cohort, age, BM blasts, and cytogenetic risk stratification were considered statistically significant, while only cytogenetic risk stratification was observed in the TARGET cohort (Table 1).

**TABLE 1:** Correlation of clinicopathologic characteristics and the 12-gene signature in acute myeloid leukemia (AML).

	**Training cohort (TCGA)**				**Validation cohort (TARGET)**			
**Characteristics**	**Total patients**	**High risk**	**Low risk**	***p*-value**	**Total patients**	**High risk**	**Low risk**	***p*-value**
Gender				0.679				0.598
Female	70	33	37		116	55	61	
Male	81	42	39		112	58	54	
Age (median)	57	63.0	51.0	**<0.001**	10	10.0	9.0	0.359
FAB Category				**0.002**				0.288
M0	13	5	8		8	6	2	
M1	35	20	15		27	12	15	
M2	35	17	18		52	20	32	
M3	13	2	11		58	26	32	
M4	33	12	21		40	25	15	
M5	17	14	3		4	2	2	
M6	2	2	0		10	6	4	
M7	3	3	0		15	7	8	
WBC (median)	15.5	12.0	20.0	0.682	36.8	31.1	49.5	0.086
BM Blasts (median)	32	17.0	39.5	**0.021**	73.2	77.5	72.0	0.546
PB Blasts (median)	71	67.0	72.0	0.254	60	56.5	62.0	0.390
Cytogenetic risk stratification				**0.001**				**<0.001**
Favorable	29	6	23		78	24	54	
Intermediate	89	47	42		118	69	49	
Adverse	31	21	10		22	14	8	

*WBC, white blood cell count; BM, bone marrow; PB, peripheral blood; TCGA, the cancer genome atlas; TARGET, therapeutically applicable research to generate effective treatments. The bold value means *P* < 0.05 with statistically significant.*

### Differentially Expressed RNA-Binding Protein-Encoding Genes Between Acute Myeloid Leukemia and Normal Tissue

**FIGURE 1:**
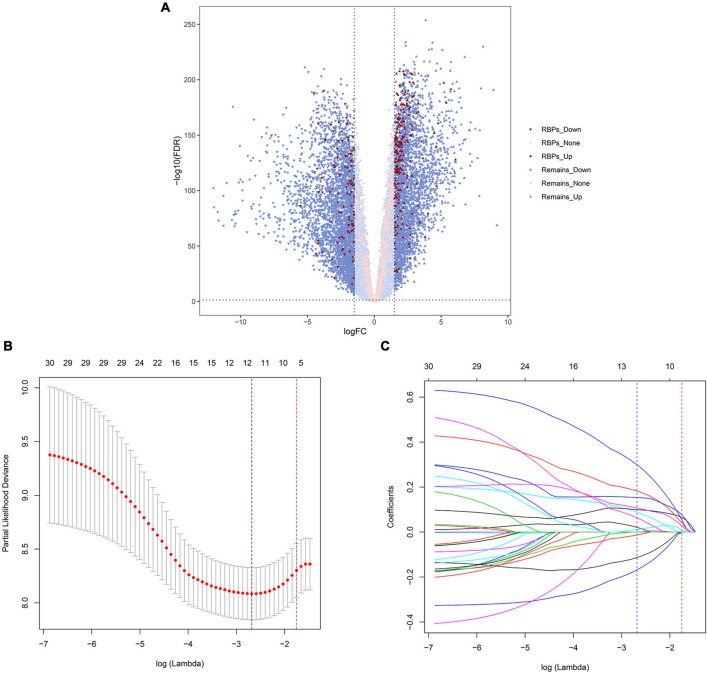
Volcano map and least absolute shrinkage and selection operator (LASSO) regression analysis of The Cancer Genome Atlas (TCGA) cohort. The volcano map shows the differential expression of genes. The up and down arrows represent upregulated and downregulated genes, respectively; red and pink represent genes with and without differential expression of RNA-binding protein (RBP)-encoding genes, respectively **(A)**. The selection of LASSO regression truncation value after cross-validation, and the blue dotted line represents the lambda corresponding to the lowest error mean. The red dotted line represents the maximum lambda corresponding to the error mean within one standard deviation of the minimum **(B)**. Prognosis-related gene selection in the LASSO–Cox regression **(C)**.

A total of 6,278 genes with significant differences in expression were identified between TCGA—AML and GTEx—blood samples, which were tested by quasi-likelihood *F*-tests and generalized linear models (glms) in *edgeR*. A total of 322 differential RBP-encoding genes met the criteria, of which 221 were upregulated and 101 were downregulated (Supplementary Table 1). Figure 1A shows a volcanic map showing this differential distribution.

### Construction and Validation of Prognostic RNA-Binding Protein Signature Model

First, a total of 33 RBP-encoding genes with significant prognostic correlation were screened for TCGA (training cohort) by univariate Cox regression analysis (Supplementary Table 2). LASSO regression analysis was then performed to further screen genes to avoid overfitting and generate a simpler, more easily interpreted model (Figures 1B,C). Finally, 12 prognostic RBP-encoding genes were selected, including LARP1B, TRNT1, SMN2, MRPL28, TRIM21, RPS19BP1, XPO6, TSR2, ISG20, HELZ2, EXOSC4, and EIF2AK4. In the training set (TCGA) and the validation set (TARGET), both differential expression analyses yield basically the same results of the 12 gene expression trends between AML and normal samples (Supplementary Figure 1). The risk score = −0.01322 × expression of LAR P1B−0.113572 × expression of TRNT1−0.16828 × expression of SMN2+0.01175 × expression of MRPL28+0.02097 × expression of TRIM21+0.06365 × expression of RPS19BP1+0.08799 × expression of XPO6+0.09969 × expression of TSR2+0.10 714 × expression of ISG20+0.15339 × expression of HELZ2+0.18212×expression of EXOSC4+0.29918×expression of EIF2AK4. The truncated value of the risk score of the patient was divided into high-risk group and low-risk group according to the median value of TCGA (training cohort) risk score of −0.085.

**FIGURE 2:**
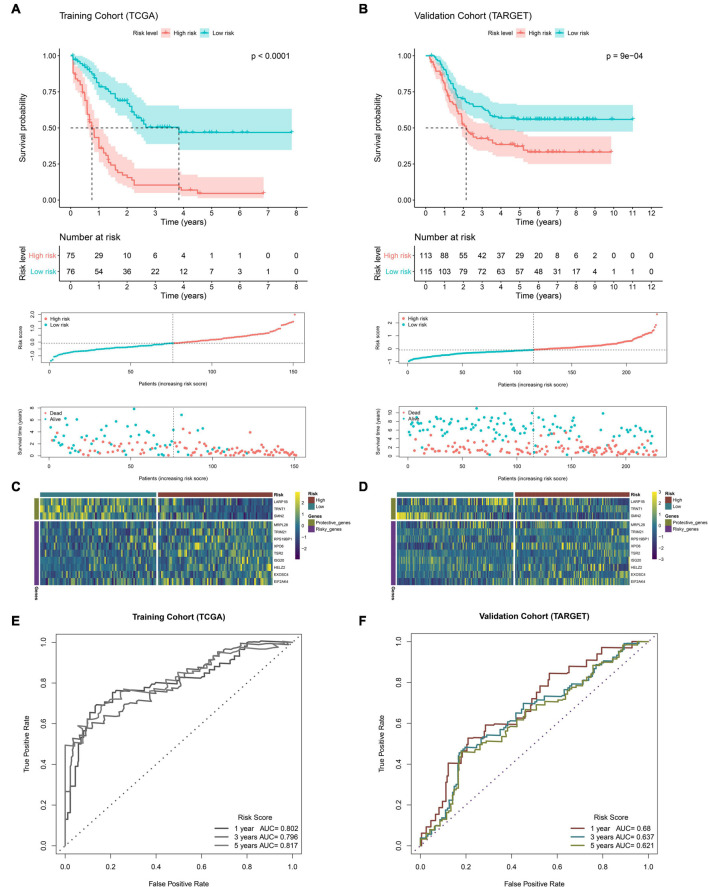
Correlation analysis based on the 12-RBP signature. Survival analysis of high and low risks separated by the median value of the TCGA cohort risk score in the TCGA **(A)** and Therapeutically Applicable Research to Generate Effective Treatments (TARGET) cohort **(B)**. The heatmap shows the expression of the 12-RBP signature at high and low risks. The green module indicates protective genes with regression coefficient less than zero, and the purple module indicates risk genes with regression coefficient greater than zero **(C,D)**. ROC curve of risk score at 1, 3, and 5 years **(E,F)**.

In the high- and low-risk groups with truncated boundaries, the survival analysis showed significant differences in both the training and validation cohort data sets (Figures 2A,B). A heat map shows the prognosis of the 12-RBP signature in the distribution of the high- and low-risk groups; one of the first module contains three protect genes (regression coefficient is less than zero), and their expression in the low-risk group is higher than in the high risk group. The second module contains nine risk genes (regression coefficient is greater than zero); their expression in the low-risk group is lower than in the high-risk group (Figures 2C,D). The areas under the ROC curve of 1-, 3-, and 5-year overall survival (OS) rates for patient risk score in the training cohort were 0.724, 0.683, and 0.650 (Figure 2E), and 0.724, 0.683, and 0.650, respectively, in the validation cohort (Figure 2F). Taken together, these results suggest that the risk score based on 12-RBP signature is a good predictor of patient prognosis.

### The Combination Model Has Good Predictive Effect

**FIGURE 3:**
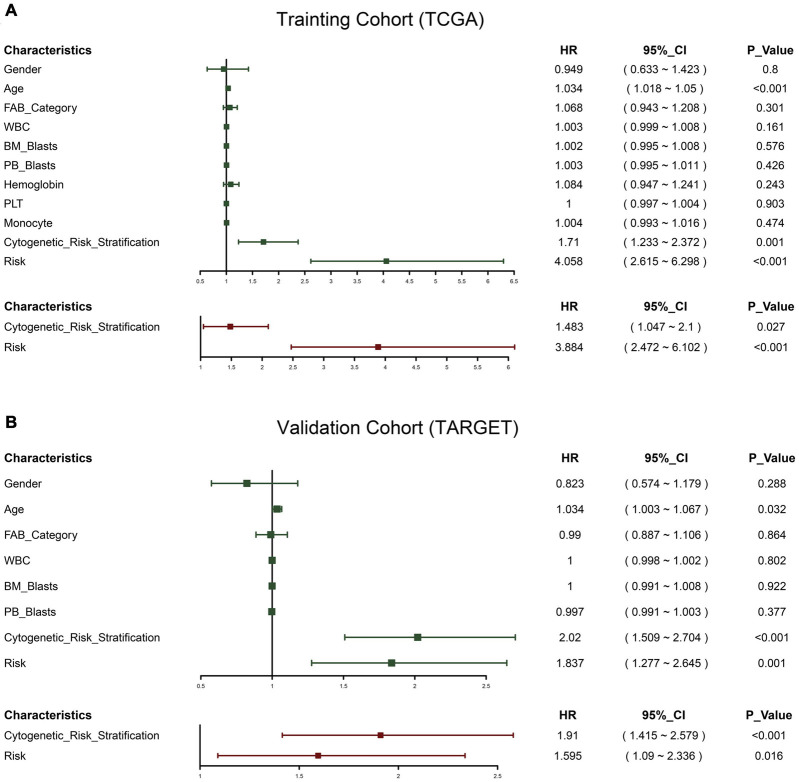
Forest plot of the univariate and multivariate Cox regression analysis in acute myeloid leukemia (AML). Green represents univariate Cox, and red represents multivariate Cox. TCGA cohort **(A)**; TARGET cohort **(B)**.

Univariate Cox was used to analyze the risk indicators constructed based on the 12-RBP signature and the clinical features including gender, age, FAB category, WBC, BM blasts, PB blasts, and cytogenetic risk stratification. It was found that age, cytogenetic risk stratification, and risk indicators were of significant prognostic value. After that, the stepwise regression optimization model was further used, and cytogenetic risk stratification and risk indicators were included in the multivariate Cox regression. It was found that these two factors had a great influence on the prognosis of patients in the two data sets, and they were both independent prognostic factors of OS (Figure 3).

**FIGURE 4:**
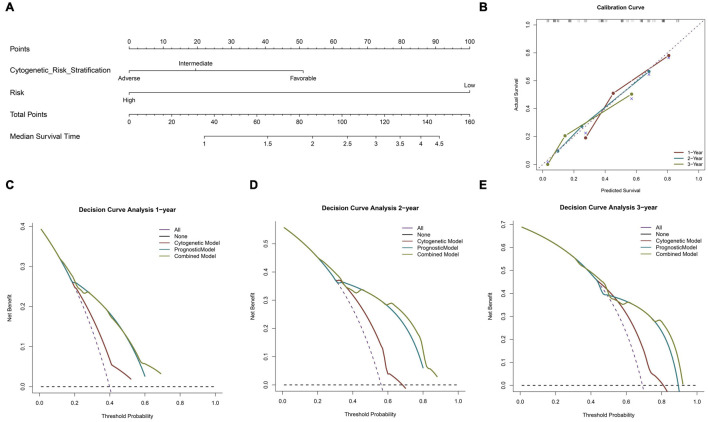
Building and validation of the nomogram predicting overall survival for AML patients. Nomogram predicting median survival time for patients with AML **(A)**. The calibration curve for predicting 1–, 2– and 3–year overall survival (OS) for patients with AML **(B)**. The decision curve analysis (DCA) curve shows the 1–, 2–, and 3–year net benefit rate of the three models. Red, blue, and green, respectively, represent the cytogenetic model, prognostic model, and combined model **(C–E)**.

A nomogram was constructed using the two previously screened indicators, and the median survival time was used to demonstrate the prognosis of patients (Figure 4A). In order to verify the accuracy of the model, we calculated the C index and drew the calibration curve. In the training cohort (TCGA), the C index of the cytogenetic model, the prognostic model, and the combined model were 0.590, 0.675, and 0.699 (Table 2), respectively. According to the results of the C index, the prognostic model constructed by us has a better prediction effect than the cytogenetic model currently used in clinical application to judge the prognosis of patients, and the prediction accuracy of the combined model is significantly higher than that of the single index model. In addition, the calibration diagram also shows that the nomogram performs well (Figure 4B). According to DCA, both the prognostic model and the combined model had a higher clinical net benefit rate than the cytogenetic model at 1, 2, and 3 years (Figures 4C–E). In Supplementary Figure 2, the prognostic model has a good degree of recognition within clinically relevant subgroups by survival analysis. Therefore, the model we analyzed is effective for the heterogeneous disease of AML.

**TABLE 2:** Comparison of the cytogenetic model, prognostic model, and combined model.

	**Training cohort (TCGA)**			**Validation cohort (TARGET)**		
**Models**	**C-index**	**95% CI**	***p*-value**	**C-index**	**95% CI**	***p*-value**
Cytogenetic model	0.59	(0.531–0.65)	–	0.62	(0.575–0.665)	–
Prognostic model	0.675	(0.629–0.721)	<0.001	0.576	(0.53–0.622)	<0.001
Combined model	0.699	(0.646–0.751)	<0.001	0.646	(0.595–0.696)	<0.001

To sum up, the prognostic model constructed by us may improve the prediction accuracy of traditional cytogenetic model and bring some net clinical benefits, which is helpful for clinical management.

### The Selection and Subsequent Analysis of RNA-Binding Protein-Encoding Gene Data Were Verified to Be Accurate

**FIGURE 5:**
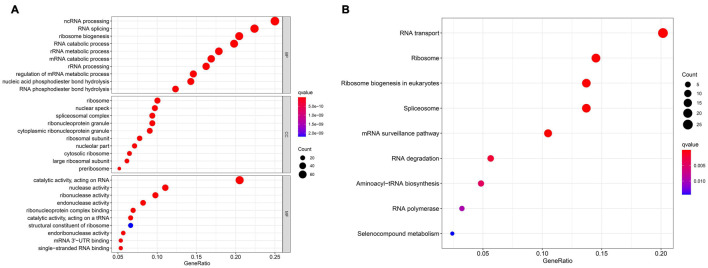
Gene Ontology (GO) enrichment analysis **(A)** and Kyoto Encyclopedia of Genes and Genomes (KEGG) pathway **(B)** of differentially expressed RBP-encoding genes.

**FIGURE 6:**
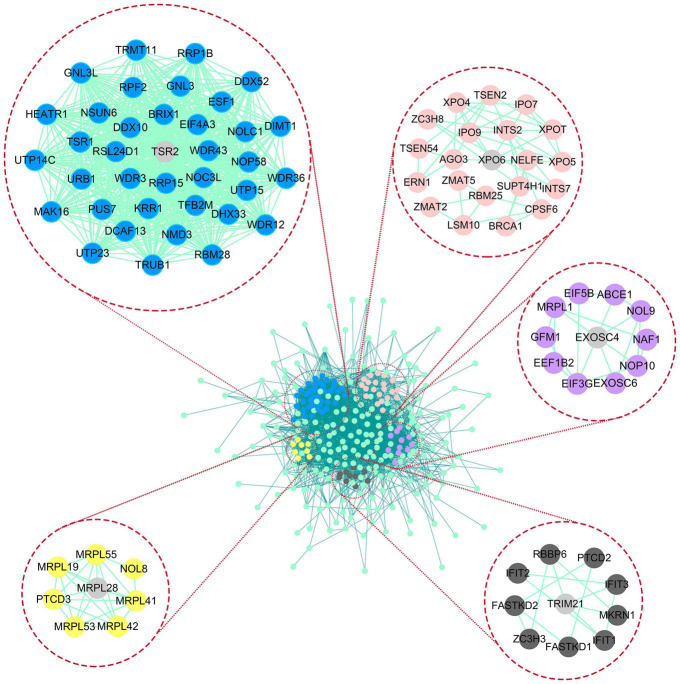
Protein–protein interaction (PPI) network and modules analysis. In the PPI network of differential genes, the matching of 12-RBP signature and core module in LASSO results was demonstrated, among which five core modules contained prognostic gene, and the gray gene represented the matching gene.

In differential expression analysis, 322 RBP-encoding genes were changed between AML and normal samples. According to the results of GO and KEGG analysis, it was found that the differentially expressed RBP-encoding genes were enriched in the processes of synthesis, regulation, transport, and translation of RNA, indicating the reliability of the source of these RBP-encoding genes (Figure 5 and Supplementary Table 3). In order to study the interaction between the differentially expressed RBP-encoding genes, we created a PPI network, which demonstrated a total of 3,038 edges and 322 nodes (Figure 6). The MODE plugin was used to identify a total of 11 core modules (Supplementary Table 4). We searched the distribution of 12-RBP signatures in the core modules and found that five of them were targeted in the five core modules of 1, 3, 5, 8, and 11. These five core modules are associated with the processes and biogenesis of ribosome, spliceosome, RNA transport, and degradation (Supplementary Figure 3 and Supplementary Table 5). In a word, evidence from pathway enrichment analysis indicating that the prognostic genes we screened had a high degree of reliability.

### Gene Set Enrichment Analyses

**FIGURE 7:**
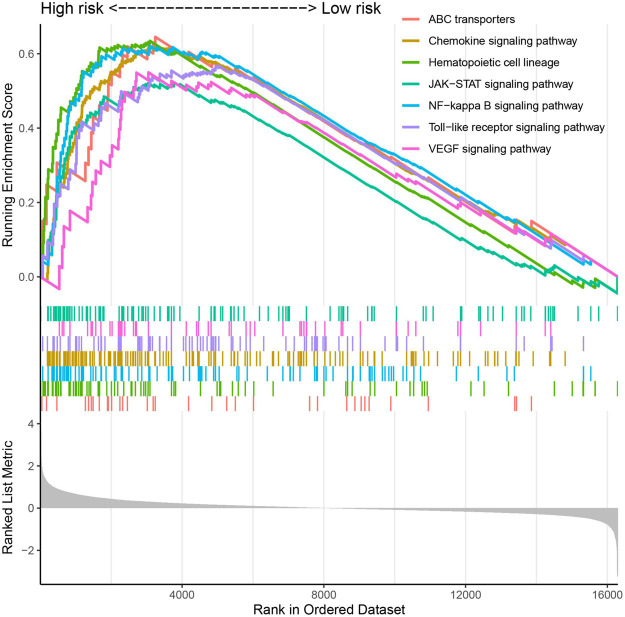
The significantly enriched KEGG pathways by Gene Set Enrichment Analyses (GSEA).

Gene set enrichment analyses analyzed 120 significantly enriched KEGG pathways between the high- and low-risk groups (Supplementary Table 6). The most enriched pathways are involved in the development of the disease, and they also accumulate into pathways associated with leukemia. The majority of the ABC transporters, hematopoietic cell lineage, NF-kappa B signaling pathway, chemokine signaling pathway, Toll-like receptor signaling pathway, VEGF signaling pathway, and JAK-STAT signaling pathway were enriched in the high-risk group (Figure 7). These biological processes are highly associated with promoting cell survival and inhibiting cell death, which play an important role in AML.

## Discussion

RNA-binding proteins are an important class of evolutionarily conserved proteins involved in regulating all aspects of RNA metabolism and functions (Nishida et al., 2017; Masuda and Kuwano, 2019). Disorders of these genes *in vitro* have been shown to cause a variety of diseases, including AML. Due to the important characteristics of RBPs, the use of RBPs to evaluate the prognosis of some cancers has achieved good results (Wang K. et al., 2019; Li et al., 2020). AML has a high recurrence rate due to the avoidance of drugs by leukemic stem cells (LSCs). PRT mode selection is the main means to prevent recurrence, but it depends on reliable prognostic markers to determine the prognosis of patients. Therefore, our systematic estimation of RBP-encoding gene changes in AML may be an important way to improve the prognosis assessment of patients with AML and may shed light on the underlying biological mechanisms.

In this study, a new and efficient prognostic feature based on the 12-RBP signature was determined based on the TCGA dataset (the training cohort), and its effectiveness was also verified in the TARGET dataset (the independent external validation sets). The group defined as high risk had poor prognosis, which was consistent in validation cohorts. Moreover, this prognostic feature is independent of other clinical factors, showing a stably high prognostic value for AML. In addition, it was considered that both the cytogenetic risk stratification and the risk indicator were significant prognostic values in AML by Cox regression. Combining the prognostic feature with the cytogenetic risk stratification, a combined model was constructed, which has higher prediction accuracy and clinical net benefit than the single model and provides a potential theoretical basis for clinical application.

In our study, 12-RBP signatures were identified and constructed the prognostic model. The expressions of LARP1B, TRNT1, and SMN2 were correlated with favorable outcomes. On the contrary, the expressions of MRPL28, TRIM21, RPS19BP1, TSR2, XPO6, ISG20, HELZ2, EXOSC4, and EIF2AK4 were involved in adverse outcomes. According to previous studies, most of the 12 RBP-encoding genes in our model are strongly cancer and other diseases. Some genes can be used as biomarkers for prognosis and diagnosis of diseases. LARP1B is a member of the evolutionary conserved family of La-related proteins (LARP) involved in RNA transcription, translation, and B-cell differentiation, which has been shown to drive tumorigenesis (Stavraka and Blagden, 2015; Lagou et al., 2018). TRNT1, as an enzyme necessary for the synthesis of the 3′-terminal CCA sequence in tRNA molecules, can lead to developmental delay, sideroblastic anemia, periodic fever, retinitis pigmentosa, B-cell immunodeficiency, and other diseases when it is abnormal (Frans et al., 2017; Slade et al., 2020). Spinal muscular atrophy (SMA) results from the absence or mutation of SMN1, plus the inability of SMN2 to compensate for the loss of SMN1 due to exon seven jumping (Lorson et al., 1999). MRPL28 encodes mitochondrial ribosomal protein, and its low expression can reduce the mitochondrial activity of pancreatic tumor cells and increase glycolysis (Chen et al., 2009). TRIM21, as a E3 ubiquitin ligase with multiple domains, regulates ubiquitination and proteasomal degradation and is responsible for the control of cell protein expression. It has been shown to regulate the cell cycle, cell proliferation and differentiation of cancer, and is a prognostic marker for hepatocellular carcinoma, breast cancer, pancreatic cancer, and lymphoma (Brauner et al., 2015; Ding et al., 2015; Nguyen and Irby, 2017; Zhou et al., 2018). As the first reported direct regulator of SIRT1 that modulates p53-mediated growth regulation, RPS19BP1 (also known as AROS) promotes survival in a panel of human cancer cell lines (Kim et al., 2007; Knight et al., 2013). TSR2 can induce apoptosis of laryngeal cancer cells by inhibiting NF-κB signaling pathway (He et al., 2018). In addition, TSR2 variation is associated with Diamond–Blackfan anemia (DBA) (Clinton and Gazda, 2009). As a nucleocytoplasmic transporter, XPO6 expression is closely related to poor prognosis of patients and is a potential prognostic biomarker for prostate cancer (Hao et al., 2016). ISG20 is involved in small nucleolar RNA maturation and ribosomal biogenesis, and controls RNA stability (Espert et al., 2006). ISG20 expression has been shown to be elevated in the course of infection and a potential biomarker for several types of cancer (Rajkumar et al., 2011; Van Tong et al., 2018; Xu et al., 2020). Katano-Toki et al. (2013) found that HELZ2 synergistic with Thrap3 enhances PPAR enzyme-mediated gene activation and plays an important role in the terminal differentiation of adipocytes. EXOSC4, one of the noncatalytic members of RNA exosome complex, is involved in RNA degradation and has been reported to promote colorectal cancer (Pan et al., 2018). EIF2AK4 induces subunit phosphorylation of the translation initiation factor eIF2, which plays an important role in oncogenesis (Koromilas, 2015). For example, EIF2AK4 participates in the EIF2AK4–EIF2alpha–ATF4 pathway, which is crucial for maintaining metabolic homeostasis in tumor cells and a potential target for tumor therapy (Ye et al., 2010; Singleton and Harris, 2012). Although the role of the 12 RBP-encoding gene expression in the pathogenesis of AML remains unclear, these genes mediate important biological processes, and their abnormalities can lead to disease. What is more, most of these genes are instructive for the prognosis in different diseases, indicating the important role of these genes.

## Conclusion

The 12-RBP signature prognostic model is an optimized biomarker for predicting the prognosis of AML patients and can be used to select postremission treatment (PRT) for AML patients, thereby reducing recurrence rates. Nomogram, a prognostic model, predicts median survival time. This study expands our current understanding of the role of RBPs in the occurrence of AML and may lay the foundation for future treatment. However, the current study also has some limitations, and we need to conduct experimental verification of the screened important genes to further verify our results.

## Data Availability Statement

Publicly available datasets were analyzed in this study. This data can be found here: https://xenabrowser.net/datapages/.

## Author Contributions

HL and YZ conceived and designed the study. NH and YH performed the data analysis. CH and HL wrote the manuscript. YH and CH revised the manuscript. All authors read and approved the final version of the manuscript.

## Conflict of Interest

The authors declare that the research was conducted in the absence of any commercial or financial relationships that could be construed as a potential conflict of interest.

## Publisher’s Note

All claims expressed in this article are solely those of the authors and do not necessarily represent those of their affiliated organizations, or those of the publisher, the editors and the reviewers. Any product that may be evaluated in this article, or claim that may be made by its manufacturer, is not guaranteed or endorsed by the publisher.

## Footnotes

1
https://toil.xenahubs.net

2
https://portal.gdc.cancer.gov/

3
https://ocg.cancer.gov/programs/target

4
https://gtexportal.org/home/

5
https://ocg.cancer.gov/programs/target/projects/acute-myeloid-leukemia

6
https://www.nature.com/articles/nrg3813#supplementary-information

7
http://www.string-db.org/

## Abbreviations

TCGA: the cancer genome atlas
GTEx: the genotype-tissue expression
TARGET: therapeutically applicable research to generate effective treatments
WBC: white blood cell count
BM: bone marrow
PB: peripheral blood
OS: overall survival
GO: gene ontology
KEGG: kyoto encyclopedia of genes and genomes
PPI: protein–protein interaction.
