# Supplementary material for: Systematic Construction and Validation of an RNA-Binding Protein-Associated Prognostic Model for Acute Myeloid Leukemia
Source: Front Genet. 2021 Sep 24;12:715840. doi: 10.3389/fgene.2021.715840 (PMC8498117; doi:10.3389/fgene.2021.715840)
Supplement: Supplementary file 1 [file Data_Sheet_1.ZIP › Supplementary_material/SupplementaryTableS4.docx]

**SupplementaryTableS4.** The 11 core modules details in PPI network construction.

| **Cluster** | **Score** | **Nodes** | | | **Edges** | **Node IDs** |
| --- | --- | --- | --- | --- | --- | --- |
| 1 | 34.778 | | 37 | 626 | | NOLC1, TRMT11, GNL3, WDR12, RRP1B, UTP15, NOP58, DDX52, BRIX1, NOC3L, DHX33, UTP14C, GNL3L, RBM28, TSR1, **TSR2**, RPF2, DCAF13, RSL24D1, KRR1, MAK16, EIF4A3, TFB2M, HEATR1, DIMT1, RRP15, WDR36, WDR43, URB1, PUS7, TRUB1, UTP23, DDX10, ESF1, NSUN6, WDR3, NMD3 |
| 2 | 20.244 | | 42 | 415 | | SART1, RPL3L, RPL22L1, PCBP1, RPL17, RPL34, RPS3A, RPLP2, DROSHA, RPP21, RPS28, RPP40, RPL36A, RPS24, UPF1, SMG1, UPF3B, RPL26, TRA2B, U2SURP, UBA52, POLR2L, POLR2J, HNRNPH2, SNRPE, SNRPD1, CD2BP2, U2AF1L4, RPS27L, NOL11, RIOK3, CASC3, MAGOHB, POLR2E, POLR2K, NUDT21, GPKOW, LSM3, CLP1, GCFC2, CCAR1, UTP20 |
| 3 | 6.571 | | 8 | 23 | | MRPL53, MRPL42, MRPL41, MRPL19, MRPL55, NOL8, PTCD3, **MRPL28** |
| 4 | 4.8 | | 6 | 12 | | ZC3HAV1L, ZC3H12C, RBM26, LENG9, HELZ, ZC3H12B |
| 5 | 4.2 | | 21 | 42 | | IPO7, NELFE, IPO9, RBM25, AGO3, ZMAT2, LSM10, XPO5, CPSF6, INTS2, **XPO6**, ERN1, INTS7, ZMAT5, TSEN54, ZC3H8, XPOT, XPO4, TSEN2, BRCA1, SUPT4H1 |
| 6 | 3.5 | | 5 | 7 | | RPL7, EIF1AX, EEF1A2, MRPL3, EEF1D |
| 7 | 3.5 | | 5 | 7 | | PNPT1, CARS2, EEF1E1, MARS2, NARS2 |
| 8 | 3.2 | | 11 | 16 | | EEF1B2, **EXOSC4**, MRPL1, NOP10, NAF1, EIF3G, NOL9, EIF5B, ABCE1, EXOSC6, GFM1 |
| 9 | 3 | | 3 | 3 | | SYNCRIP, MATR3, SSB |
| 10 | 3 | | 3 | 3 | | LUC7L3, PRPF4B, TCERG1 |
| 11 | 2.889 | | 10 | 13 | | **TRIM21**, PTCD2, FASTKD2, RBBP6, IFIT3, FASTKD1, IFIT1, MKRN1, IFIT2, ZC3H3 |
