# Supplementary material for: Systematic Construction and Validation of an RNA-Binding Protein-Associated Prognostic Model for Acute Myeloid Leukemia
Source: Front Genet. 2021 Sep 24;12:715840. doi: 10.3389/fgene.2021.715840 (PMC8498117; doi:10.3389/fgene.2021.715840)
Supplement: Supplementary file 1 [file Data_Sheet_1.ZIP › Supplementary_material/SupplementaryTableS5.docx]

**SupplementaryTableS5.** The KEGG enriched pathways of five core modules.

| **Module** | **Term** | **GeneRatio** | ***P* value** | **Count** |
| --- | --- | --- | --- | --- |
| module 1 | hsa03008: Ribosome biogenesis in eukaryotes | 78.60% | 6.06E-19 | 11 |
| module 3 | hsa03010: Ribosome | 100.00% | 3.77E-04 | 2 |
| module 5 | hsa03040: Spliceosome | 28.60% | 6.46E-03 | 2 |
|  | hsa03013: RNA transport | 28.60% | 0.010189 | 2 |
| module 8 | hsa03018: RNA degradation | 50.00% | 5.58E-04 | 2 |
| module 11 | hsa05322: Systemic lupus erythematosus | 50.00% | 0.033358 | 1 |
|  | hsa05160: Hepatitis C | 50.00% | 0.038458 | 1 |
