# Supplementary figures and images for: Systematic Construction and Validation of an RNA-Binding Protein-Associated Prognostic Model for Acute Myeloid Leukemia
Source: Front Genet. 2021 Sep 24;12:715840. doi: 10.3389/fgene.2021.715840 (PMC8498117; doi:10.3389/fgene.2021.715840)

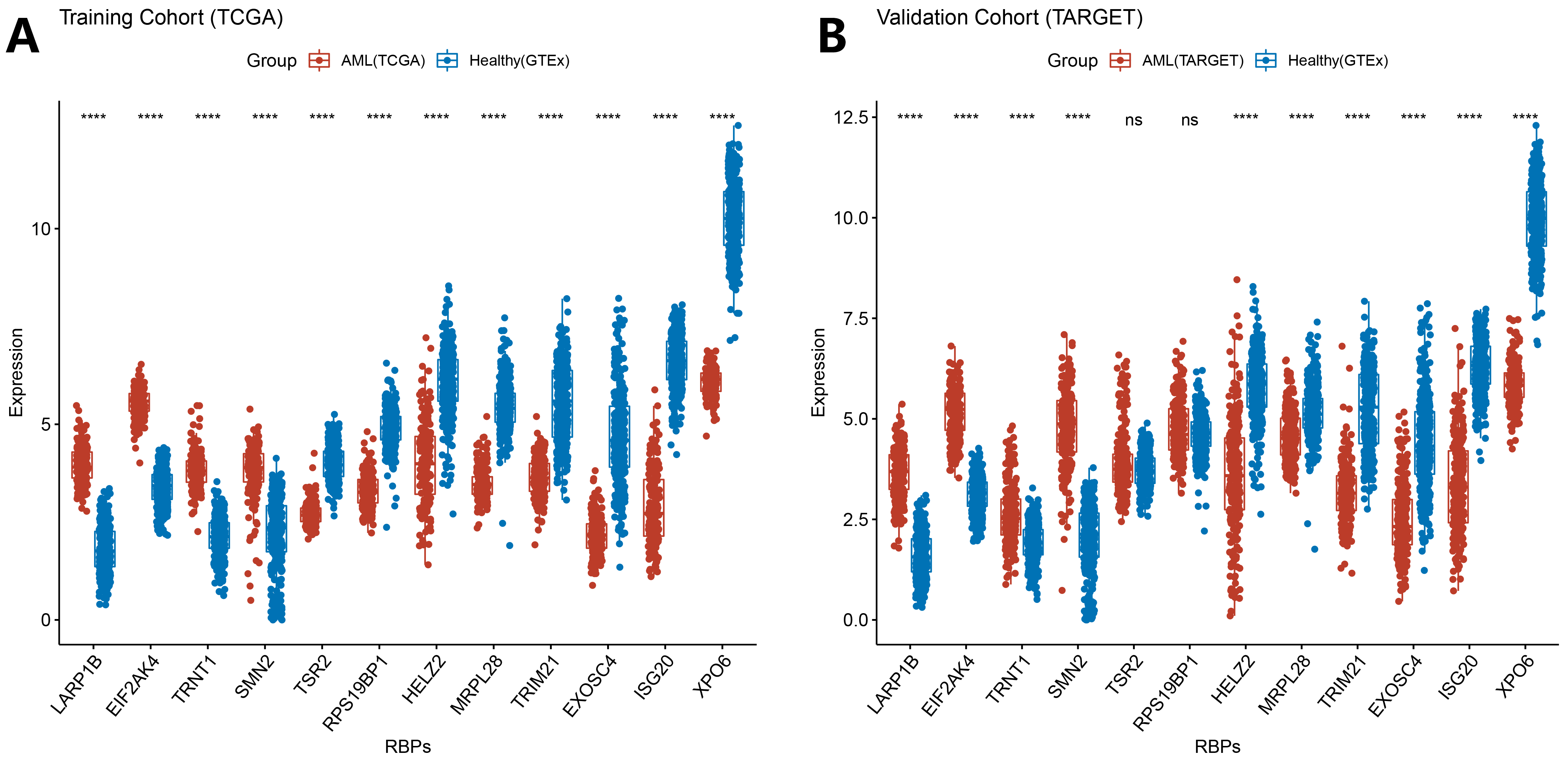

Supplement: Supplementary file 1 [file Data_Sheet_1.ZIP › Supplementary_material/SupplementaryFigureS1.tif]

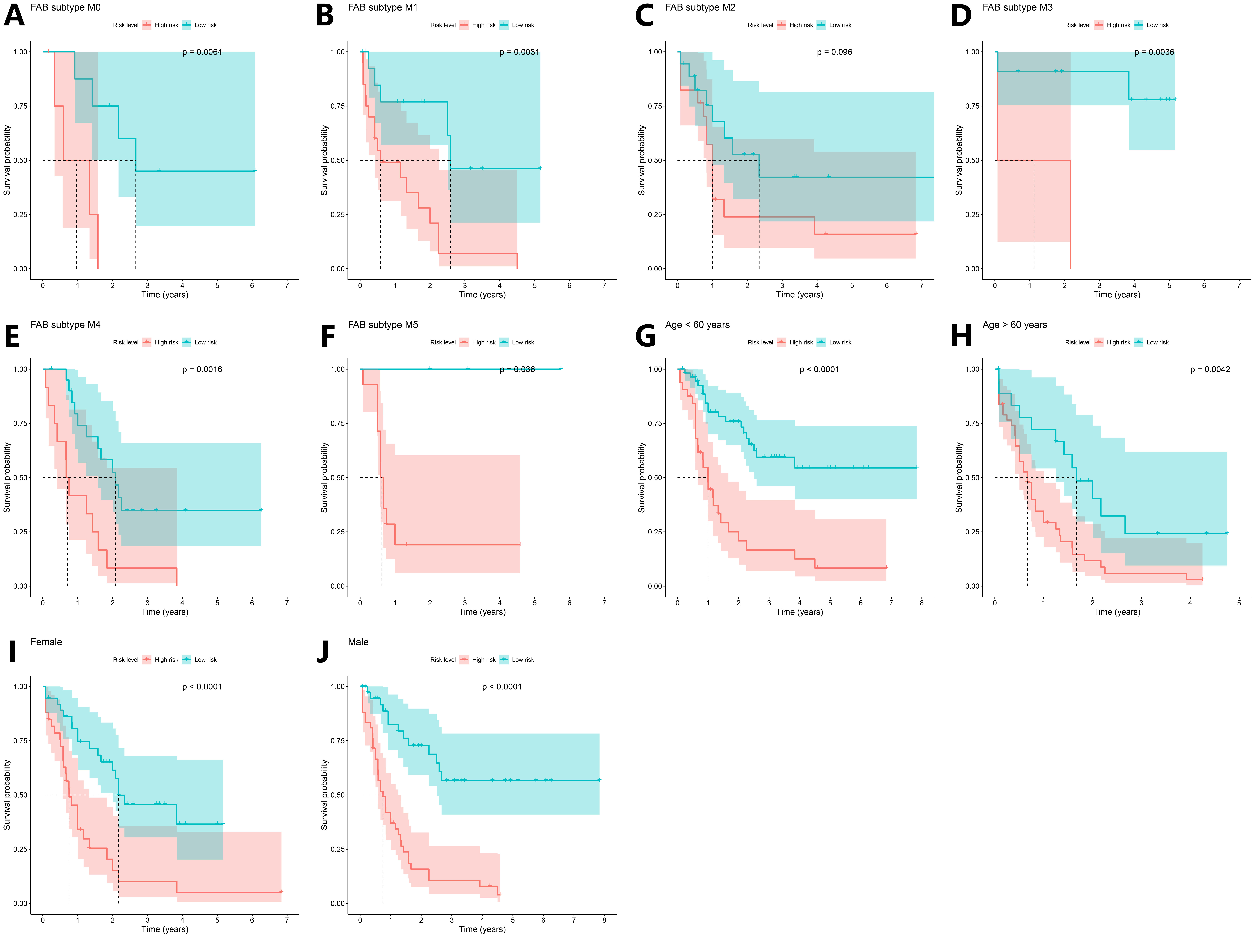

Supplement: Supplementary file 1 [file Data_Sheet_1.ZIP › Supplementary_material/SupplementaryFigureS2.tif]

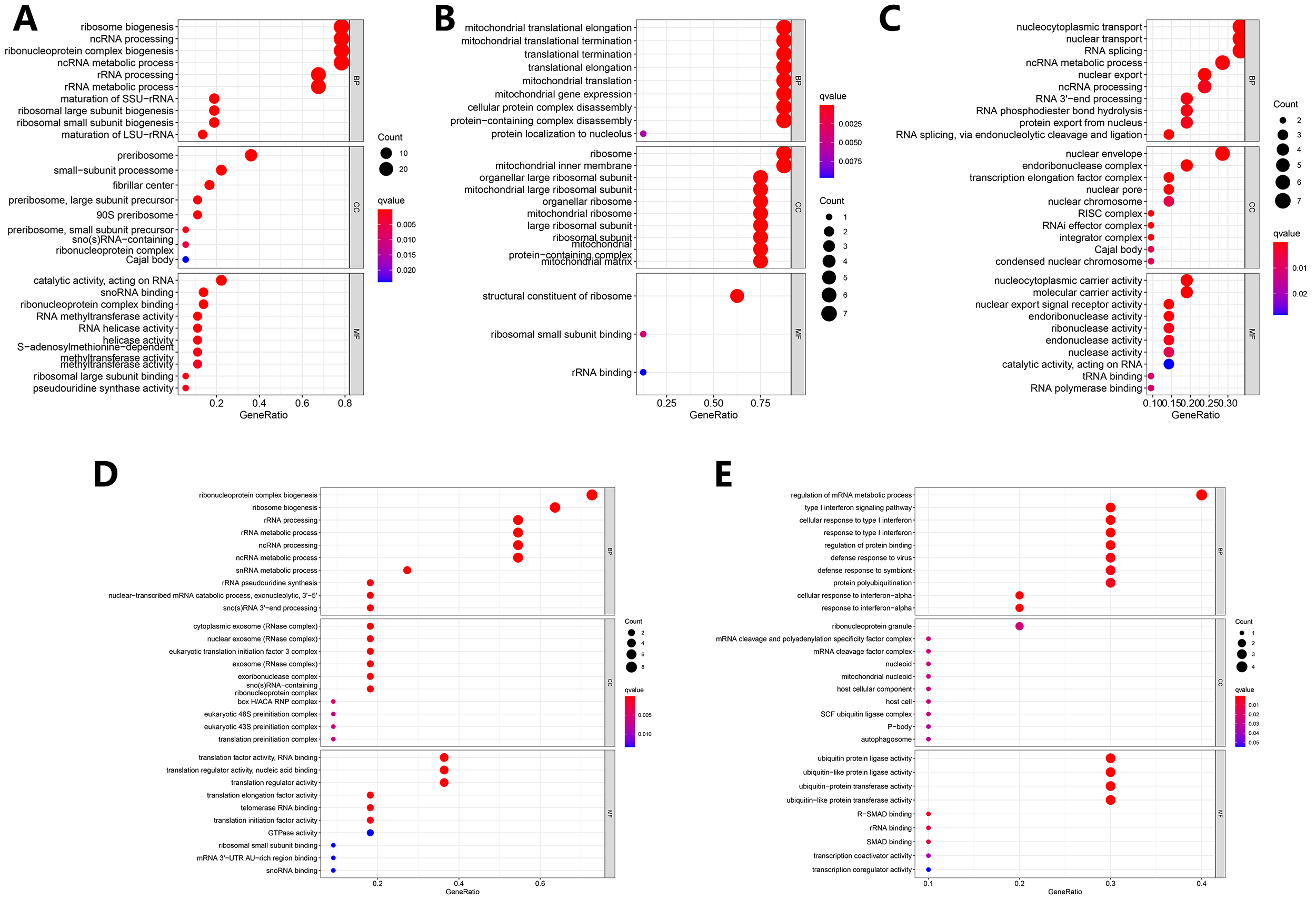

Supplement: Supplementary file 1 [file Data_Sheet_1.ZIP › Supplementary_material/SupplementaryFigureS3.tif]
